# “Implications of cost-sharing for observation care among Medicare beneficiaries: a pilot survey”

**DOI:** 10.1186/s12913-019-3982-8

**Published:** 2019-03-07

**Authors:** Jennifer N. Goldstein, J. Sanford Schwartz, Patricia McGraw, LeRoi S. Hicks

**Affiliations:** 1 Department of Medicine & The Value Institute, Christiana Care Health System, 4755 Ogletown-Stanton Rd, Ammon Education Center Suite 2E70, Newark, DE 19713 USA; 2 Division of General Internal Medicine, University of Pennsylvania, 1203 Blockley Hall, 423 Guardian Drive University, Philadelphia, PA 19104 USA; 3 Department of Medicine & The Value Institute, Christiana Care Health System, 4755 Ogletown-Stanton Rd, Ammon Education Center Suite 2C50, Newark, DE 19713 USA

**Keywords:** Healthcare policy, Poverty, Public policy, Access to and utilization of healthcare, Disparities

## Abstract

**Background:**

Medicare beneficiaries hospitalized under observation status have significant cost-sharing responsibilities under Medicare Part B. Prior work has demonstrated an association between increased cost-sharing and health care rationing among low-income Medicare beneficiaries. The objective of this study was to explore the potential impact of observation cost-sharing on future medical decision making of Medicare beneficiaries.

**Methods:**

Single-center pilot cohort study. A convenience sample of Medicare beneficiaries hospitalized under observation status care was surveyed.

**Results:**

Out of 144 respondents, low-income beneficiaries were more likely to be concerned about the cost of their observation stay than higher-income respondents (70.7% vs29.3%, *p* = 0.015). If hospitalized under observation status again, there was a trend among low-income beneficiaries to request completion of their workup outside of the hospital (56.3% vs 43.8%), and to consider leaving against medical advice (AMA) (100% vs 0%), though these trends were not statistically significant (*p* = 0.30).

**Conclusion:**

The results of this pilot study suggest that low-income Medicare beneficiaries hospitalized under observation status have greater concerns about their cost-sharing obligations than their higher income peers. Cost-sharing for observation care may have unintended consequences on utilization for low-income beneficiaries. Future studies should examine this potential relationship on a larger scale.

**Electronic supplementary material:**

The online version of this article (10.1186/s12913-019-3982-8) contains supplementary material, which is available to authorized users.

## Background

Observation status or “hospital outpatient status” is a classification for Medicare beneficiaries that are billed as outpatients for a hospitalization. Whereas hospital inpatients are billed through Medicare Part A, observation patients are billed through Medicare Part B, which can result in higher out-of-pocket costs [1]. Since 2013, The Centers for Medicare and Medicaid Services (CMS) has defined observation patients by the 2-Midnight Rule, which stipulates that patients with an anticipated length of stay of < 2 midnights be designated as observation status while those anticipated to require ≥2 midnights be designated as inpatients, regardless of clinical status. Prior to this, observation status was determined by clinical criteria.

Observation visits have been on the rise for over a decade. Between 2006 and 2010, the use of observation stays increased 70%, largely as a result of penalties that CMS was imposing on hospital systems under the Recovery Audit Contractor Program for inappropriately billing for short-stay admissions [2, 3]. The introduction of the 2-Midnight Rule has increased the use of observation stays by another 8% [4]. It is estimated that approximately 25% of adult general medicine hospitalizations are observation visits [5].

Observation patients and inpatients have different cost-sharing responsibilities. Under Medicare Part A, inpatients pay a substantial deductible ($1364 in 2019), which covers most of the care in the hospital, 21 days in a skilled nursing facility if needed after the hospital stay, and hospital readmissions within 60 days of discharge [6]. Observation patients have a lower deductible, ($185), and higher cost-sharing for hospital services (20%) under Medicare Part B. They also are billed for “self-administered medications” which are medications that are not directly related to the primary diagnosis for the hospitalization [7]. Unlike Part A, Part B does not cover the cost of rehospitalizations, so patients can face cumulative out-of-pocket expenses for repeat observation stays. A prior study of Medicare claims demonstrated that the median out-of-pocket costs per observation stay are $448.94 [8] and that cumulative out-of-pocket expense for re-hospitalizations for observation stays exceeds that of the Medicare Part A deductible for over 25% of beneficiaries [9].

Our prior work has demonstrated that low-income patients are at higher risk for higher utilization of observation care and higher out-of-pocket costs related to this care compared to higher income patients [10]. Additionally, there is an association between cost-sharing and healthcare rationing among low-income Medicare beneficiaries [11, 12]. As more beneficiaries are exposed to Part B cost- sharing for their hospitalizations it is unclear whether this will impact beneficiary attitudes and behavior surrounding observation care. Our objective was to explore whether cost-sharing for observation care could impact future medical decision-making related to such care among low-income versus higher-income Medicare beneficiaries.

## Methods

This is a pilot study of a convenience sample of Medicare beneficiaries hospitalized under observation status to Christiana Care Health System (CCHS) from January 4, 2016-May 19, 2016.

### Sample/population

Eligible patients had traditional Medicare as their primary form of insurance and were hospitalized under the care of one of the non-teaching hospitalist services at either Wilmington Hospital, a 241 bed community hospital in Wilmington, DE or Christiana Hospital, a 913 bed tertiary care facility in Newark, DE. Patients were excluded if they were younger than 18 years of age, ICU or step-down admissions, direct admissions from home, transfers from other services, on the teaching service, listed as “comfort care only”, or as a “confidential” patient. Designated family members could answer questions at the patients request or if the patient was confused or had a history of significant cognitive impairment. The CCHS health informatics team sent a daily list of eligible patients to the principal investigator and research nurse. All eligible patients were approached during weekday, workday hours. The convenience sample was obtained based on patient availability, willingness, and ability to complete the survey.

### Survey instrument

A 23-item survey was administered in person by a trained, unblinded, research assistant, within 24 h of their hospitalization. The first 3 questions addressed awareness and understanding of observation status and policies and were based on language from a CMS patient information pamphlet [7].

Questions 4–9 and 11–16, were taken directly from the 2014 National Health Interview Survey (NHIS) and addressed rationing of health services (mental health, vision care, dental, and specialist and primary care) and prescriptions due to cost in the previous 12 months. These questions were chosen to examine baseline concerns related to health care cost-sharing. The original NHIS questions had 4 responses (Yes, No, Refused, Don’t Know); We modified this slightly by offering the responses: Yes, No, Refused, N/A. Any patient who had a positive (yes) response to any of the NHIS questions related to rationing was coded has having health-related cost concerns. Question 10, was a modified version of NHIS question AAU.113 regarding concern about paying for the current hospitalization [13], and was analyzed as a dichotomous variable. Questions 17–21 were demographic in nature (age, race, ethnicity, self-reported income,). Income above or below 200% of the federal poverty line was assessed (approximately 31 K for a family of 2) to define low-income patients [14] (Additional file 1).

The last 2 questions assessed whether comprehension of Medicare observation policy could impact future health decision-making. Question 22 asked whether the patient would inquire about observation status on a future admission and had a yes/no response. Question 23 asked whether the patient would: 1) stay for the care prescribed in the hospital 2) ask the provider to arrange for services as an outpatient or 3) leave against medical advice (AMA) if hospitalized under observation status again (Additional file 1). Prior to asking questions 22 and 23, the research assistant reviewed the answers to questions 2&3 that addressed specifics of the Medicare observation policy to ensure that the patient understood the cost-sharing implications. Data regarding date of birth, age, insurance and hospitalist group were obtained from the electronic medical record. Race and ethnicity data were obtained from the electronic medical record for non-respondents. All responses were recorded on a paper copy and entered into REDCap software.

### Analysis

Descriptive statistics were reported for demographics, baseline health care rationing behaviors, and understanding of observation policies. Chi square was used to determine sociodemographic differences between responders and non-responders and to determine whether income level was associated with a history of health care rationing and cost concerns and future decision-making related to observation care. All statistical analysis was performed using STATA13, College Station, TX. All patients and/or the designated family member signed informed consent. The Christiana Care Health System Institutional Review Board approved this study.

### Sample size

Based on prior work that demonstrated a 3–4% reduction in health care utilization after introduction of cost-sharing measures [11, 12], we estimated that 686 interviews were needed to achieve 80% power for a two-tailed test with 95% confidence. For this pilot study, we had resources to perform interviews for 6 months.

## Results

There were 466 patients eligible for the study, and 168 (38%) were either missed or discharged prior to being approached for enrollment. Of those approached, 127 had a medical or cognitive impairment that prohibited completion and 11 refused. There were 160 patients who completed interviews, and of these, 150 were completed by the patient, and 10 by designated family members. Post-hoc analysis determined that 16 respondents did not have traditional Medicare as their primary insurance and were excluded, leaving 144 for analysis (Additional file 2: Figure S1). Respondents were more likely to be younger than non-respondents (*p* = 0.008) but were otherwise similar (Table 1).

**Table 1:** Characteristics of Survey Respondents and Non-Respondents

	Not completed (*n* = 306)	Completed (*n* = 144)	*p*-value
	*n*(%)	*n*(%)	
Age			
65 years old or younger	49 (16.0)	38 (26.4)	0.008
66–75	89 (29.1)	47 (32.6)	
76 or older	168 (54.9)	59 (40.9)	
Sex			
Female	176 (57.5)	93 (64.6)	0.154
Race			
Caucasian	202 (66.0)	193 (64.6)	0.955
African American	86 (28.1)	42 (29.2)	
Other	18 (5.9)	9 (6.3)	
Ethnicity			0.207
Hispanic	9 (3.0)	1 (0.7)	
Level of Education			
Less than high school	–	14 (9.7)	
High school/GED	–	53 (36.8)	
Some College/Trade school	–	20 (13.9)	
2 year College/Associates Degree	–	15 (10.4)	
4 year college	–	21 (14.6)	
Master’s Degree	–	10 (6.9)	
Doctorate	–	3 (2.1)	
Refused	–	8 (5.6)	
Supplemental Insurance (*n* = 143)	–		
Commercial	–	109 (76.2)	
Medicaid	–	10 (9.2)	
None		24 (16.8)	
Annual Income (*n* = 144)			
Below $31,000^a^	–	66 (45.8)	
Greater than or equal to $31,000	–	55 (38.2)	
Refused		23 (16)	
Cost-Sharing Questions	True *n*(%)	False *n*(%)	Refused
“As an observation patient you may need to pay more for tests than an inpatient”	43 (29.9)^b^	48 (33.3)	53 (36.8)
“As an observation patient Medicare will pay for a nursing home if you need it”	73 (50.7)	24 (16.7)^b^	47 (32.6)

^a^200% Federal Poverty line for household of 2; Source: US Department of Health and Human Services https://aspe.hhs.gov/poverty-guidelines

^b^Correct response; 11/125 answered both questions correctly

Close to half (45.8%; 66/144) of respondents were low-income by self-report. Among those who responded, approximately 17% (16.8%;24/143) did not have supplemental insurance, 37.1% (36/97) delayed seeking care due to cost concerns and 42.5% (51/120) rationed prescriptions due to cost in the previous 12 months.

Over half of respondents (52.8%; 76/144) were aware of their observation status but only 8.8% (11/125) answered both questions related to observation policies correctly (Table 1). Approximately 1/3 (34.4%; 41/119) of respondents expressed concern about paying for their observation stay. Low-income respondents were more likely to be concerned about the cost of their stay than higher-income respondents (70.7% vs 29.3%, *p* = 0.015) (Table 2).

**Table 2:** Association between Income level, Cost concerns, and Future Utilization

	Self-Reported Household Income			
	Total	<$31,000	>$31,000	
	*n*(%)	*n*(%)	*n*(%)	*p*-value
Cost Concerns				
“Regarding this hospital stay, how worried are you that you will be able to pay your medical bills?” (*n* = 119; 25 refused)				0.015
Worried	41 (34.4)	29 (70.7)	12 (29.3)	
Future Health Care Decision Making				
“If you came to the hospital in the future, would you ask if you were admitted as an observation patient?” (*n* = 93, 51 refused)				0.476
Yes	76 (81.7)	43 (56.6)	33 (43.4)	
“What would you do if you were admitted as an observation patient in the future?” (*n* = 102, 12 refused)				0.300
Stay for the care	68 (72.3)	33 (48.5)	35 (51.5)	
Ask provider to arrange services as outpatient	32 (34)	18 (56.3)	14 (43.8)	
Leave Against Medical Advice	2 (2.1)	2 (100)	0	

### Potential impact of observation cost-sharing on future observation utilization

The majority of respondents (72.3%; 68/102) stated that they would stay for the duration of the hospitalization if they were hospitalized under observation status in the future. However, approximately 1/3 (34%; 32/102) would request that their work-up be performed as an outpatient. Low-income beneficiaries were less likely to stay for the care they needed (48.5% vs 51.5%), more likely to request outpatient completion of their workup (56.3% vs 43.8%), and more likely to leave AMA if hospitalized under observation status again (100% vs 0%), though these trends were not statistically significant (Table 2).

## Discussion

In this pilot study of 144 Medicare beneficiaries hospitalized under observation status, most patients were aware of their status, but very few understood the cost-sharing implications. Low-income beneficiaries were more concerned about the cost of their hospital stay and were more likely to consider requesting outpatient completion of their work-up or leaving AMA compared to higher-income beneficiaries. Our findings imply opportunities for early or pre-admission education of Medicare beneficiaries regarding observation status, and consideration of the potential unintended consequences of the current policies.

From a legal standpoint, Medicare beneficiaries are required to be made aware of their cost-sharing responsibilities under observation status [15]. However it is still unclear how the cost-sharing itself may impact future behavior. In the outpatient setting, prior work has demonstrated that low-income patients are more likely to defer, delay or ration care if their cost-sharing responsibilities increase [11, 12, 16]. This may be associated with higher hospital utilization [12].

Although the cost-sharing responsibilities related to observation care have been in place for decades, the number of patients exposed has increased [3, 17]; as of 2010, 9.3% of beneficiaries were hospitalized under observation status [1] and the number is expected to rise [18]. Medicaid can provide significant protections against out-of-pocket costs as can supplemental insurance programs, such as Medigap; However, many patients who are eligible for Medicaid do not enroll [19] and Medigap programs, can be costly [20, 21]. Nationally, as was reflected in our study sample, approximately 14% of beneficiaries, representing over 6,700,000 individuals are not enrolled in supplemental insurance plans [21]. These patients often do not qualify for Medicaid but cannot afford supplemental coverage and are at greatest risk for high out-of-pocket costs [21].

In the context of our limited study, we found that low-income beneficiaries were more likely to express concern about the cost of their hospital stay and were more likely to consider outpatient work up or leaving AMA if hospitalized under observation status in the future compared to higher-income beneficiaries. We also found that many beneficiaries already ration health care services, a sign of significant financial strain [11, 12, 16]. As observation hospitalizations continue to rise, it will be important to proactively identify and support beneficiaries at risk for significant health care cost burden. Future research should prospectively evaluate health utilization behaviors of patients with significant out-of-pocket costs related to observation care.

### Limitations

There are several important limitations to our study. We surveyed a convenience sample of beneficiaries, which limits the generalizability of our findings. Additionally, a substantial number of eligible patients were excluded because they were either confused/demented for discharged within 24 h. This could have biased our sample in favor of sicker observation patients, without cognitive impairment, which also limits the generalizability of our findings. In the context of this pilot study we were underpowered, and were only able to detect a potential signal regarding the direction of our findings. However, our initial sample size calculation was based on an estimate that 3–4% of patients would ration observation care due to concerns related to cost-sharing, and our study found that a substantially higher proportion would consider forgoing care related to their observation stay after learning about cost-sharing obligations (34% would request outpatient work-up and 2% would leave AMA). Assuming our sample was generalizable, a larger patient sample could further strengthen the association that we found. However, a large study using a more representative patient sample could potentially result in a smaller effect size. An additional limitation was that the questions that addressed patient understanding of observation policy and the potential impact on future decisions were not validated. We could not identify any validated surveys that addressed these questions. However, we tested our survey on 10 Medicare observation patients for comprehension and not verified.

## Conclusion

As exposure to cost-sharing for observation care increases, its impact on the health behaviors, financial, and physical wellness of our most vulnerable beneficiaries remains unclear. Future prospective studies should examine this issue on a larger scale.

## Additional files

Additional file 1: Appendix 1. Patient Interview. (PDF 48 kb)

Additional file 2: **Figure S1.** Study Sample. Characteristics of respondents and non-respondents. (TIF 58 kb)

## Abbreviations

AMA: Against Medical Advice
CCHS: Christiana Care Health System
CMS: Centers for Medicare and Medicaid Services
NHIS: National Health Interview Survey
