# Supplementary material for: “Implications of cost-sharing for observation care among Medicare beneficiaries: a pilot survey”
Source: BMC Health Serv Res. 2019 Mar 7;19:149. doi: 10.1186/s12913-019-3982-8 (PMC6407198; doi:10.1186/s12913-019-3982-8)
Supplement: Supplementary file 1 — Appendix 1. Patient Interview. (PDF 48 kb) [file 12913_2019_3982_MOESM1_ESM.pdf]

Interview

Study ID ______________________________

Patient Interview

You have been admitted under observation status. Observation status lets your doctor do tests or give you treatments. This helps your doctor decide if you can go home soon or if you need to stay longer to get more treatment to get better. If you do need more treatments you may be changed to an in-patient.

1) Did anyone tell you that you are an Observation patient?
- ◯ Yes
- ◯ No
- ◯ Refused

2) True or False As an Observation patient you may need to pay more for tests and studies than an inpatient?
- ◯ True
- ◯ False
- ◯ Refused

3) Some people need extra care after they leave the hospital.

True or false: As an Observation patient Medicare will pay for you to go to a nursing facility if you need it.
- ◯ True
- ◯ False
- ◯ Refused

The next questions are about your medical care.

There are many reasons people delay getting medical care

During the past 12 months, was there any time when you needed any of the following but couldn't afford it?

4) Prescription medicines
- ◯ Yes
- ◯ No
- ◯ Refused
- ◯ NA

(NA = does not take meds)

5) Mental health counseling (MHC)
- ◯ Yes
- ◯ No
- ◯ Refused
- ◯ NA

(NA = MHC not needed)

6) Dental Care, including check-ups
- ◯ Yes
- ◯ No
- ◯ Refused
- ◯ NA

(NA = does not see a dentist)

7) Eyeglasses

◯ Yes
◯ No
◯ Refused
◯ NA
(NA = does not wear glasses)

8) To see a specialist

◯ Yes
◯ No
◯ Refused
◯ NA
(NA = no need to see a specialist)

9) Follow up care

◯ Yes
◯ No
◯ Refused
◯ NA
(NA = no follow up needed)

Hospital Stay

[Regarding this hospital stay,]

10) How worried are you that you will be able to pay your medical bills?

◯ Very worried
◯ Somewhat worried
◯ Not at all worried
◯ Refused

During the past 12 months, were any of the following true?

11) You skipped medication doses to save money

◯ Yes
◯ No
◯ Refused
◯ NA
(NA = not taking any meds)

12) You took less medicine to save money

◯ Yes
◯ No
◯ Refused
◯ NA
(NA = not taking any meds)

13) You delayed filling a prescription to save money

◯ Yes
◯ No
◯ Refused
◯ NA
(NA = not taking any meds)

14) You asked your doctor for a lower cost medication to save money

◯ Yes
◯ No
◯ Refused
◯ NA
(NA = not taking any meds)

15) You bought a prescription drug from another country to save money

◯ Yes
◯ No
◯ Refused
◯ NA
(NA = not taking any meds)

16) You used alternative therapies to save money

- ◯ Yes
- ◯ No
- ◯ Refused

Basic information about you.

17) What is the highest level of education you have completed?

- ◯ Less than high school
- ◯ High School/GED
- ◯ Some college/Trade school
- ◯ 2 Year college/Associates degree
- ◯ 4 Year college degree
- ◯ Master's degree
- ◯ Doctorate Degree
- ◯ Refused
- ◯ Other

other education? ______________________________

18) What is your annual household income?

- ◯ Greater than or equal to $31,000
- ◯ Less than $31,000
- ◯ Refused

19) What ethnicity best describes you?

- ◯ Hispanic or Latino
- ◯ Not Hispanic or Latino
- ◯ Refused

20) What race best describes you?

- ◯ American Indian/Alaska Native
- ◯ Asian
- ◯ Black or African American
- ◯ Native Hawaiian or Other Pacific Islander
- ◯ White
- ◯ More Than One Race
- ◯ Unknown / Not Reported

Lastly, I would like to go back to some of the first questions you answered:

[Go over questions 2&3 and give them the correct responses]

21) If you came to the hospital in the future, would you ask whether you are admitted as an observation patient or an inpatient?

- ◯ Yes
- ◯ No
- ◯ Refused

22) If you are admitted as an observation patient in the future, would you:

- ◯ Stay for the care that you need as an observation patient
- ◯ Leave the hospital before the doctor discharges you (which is considered against medical advice)
- ◯ Ask the provider (doctor, nurse practitioner, resident, physician assistant, etc.)/staff if they can arrange your treatments (i.e.: x-rays, physical therapy, infusions, dressing changes, etc.) to be done outside of the hospital.
- ◯ Refused

General Comments

Comments: ________________________

Last Name: Person obtaining Consent/Interview ________________________
