# Supplementary figures and images for: “Implications of cost-sharing for observation care among Medicare beneficiaries: a pilot survey”
Source: BMC Health Serv Res. 2019 Mar 7;19:149. doi: 10.1186/s12913-019-3982-8 (PMC6407198; doi:10.1186/s12913-019-3982-8)

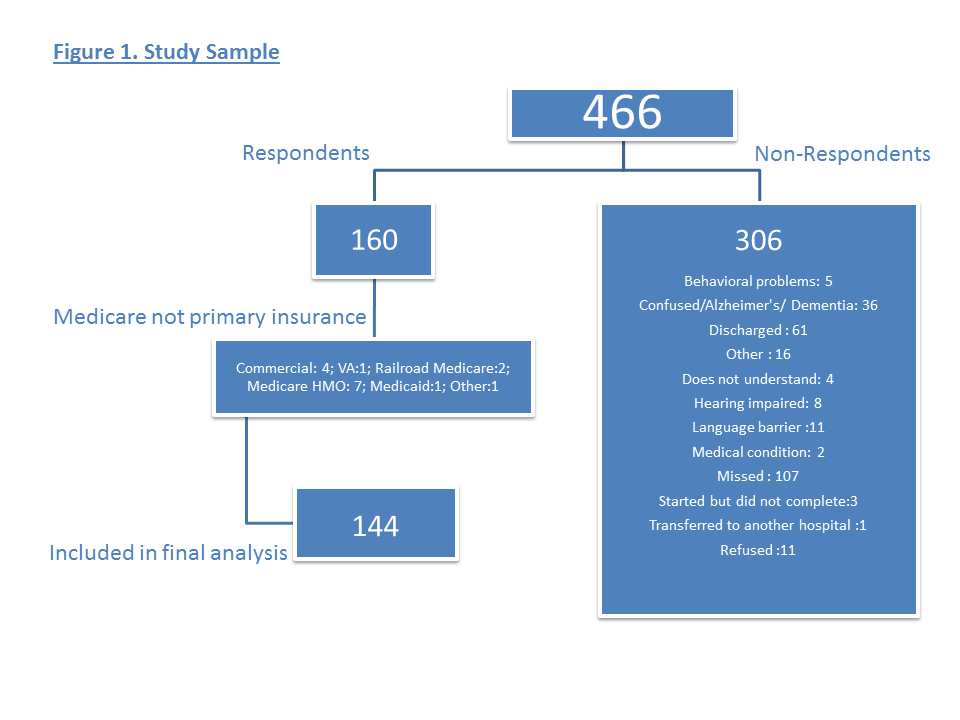

Supplement: Supplementary file 2 — Figure S1. Study Sample. Characteristics of respondents and non-respondents. (TIF 58 kb) [file 12913_2019_3982_MOESM2_ESM.tif]
